# National health technology assessment in Turkiye after a decade: are key principles followed?

**DOI:** 10.1017/S0266462323000466

**Published:** 2023-07-24

**Authors:** Tuba Saygın Avşar, Hasan Hüseyin Yıldırım

**Affiliations:** 1 Department of Applied Health Research, University College London, London, UK; 2 Department of Health Management, University of Health Sciences, Ankara, Turkiye

**Keywords:** economic evaluation, health policy, health technology assessment, health economics, HTA, low- and middle-income countries (LMIC), priority setting, Turkiye HTA

## Abstract

**Background:**

Health technology assessment (HTA) is growing in low- and middle-income countries (LMICs) to ensure optimal use of limited resources. However, the impact of HTAs on decision making in LMICs has been limited. The study aimed to provide an overview of Turkiye’s progress since establishing the first HTA agency in 2012.

**Methods:**

The web sites of three national HTA agencies in Turkiye were searched for HTA guidelines and national HTA reports. The HTA guidelines were assessed by two researchers independently against the key principles of HTA developed by Drummond et al., and the HTA reports against the national guidelines.

**Results:**

The study included one HTA guideline and eight national HTA reports. The guideline included very limited technical guidance. Compliance with the principles was poor to moderate, and significant methodological limitations were identified. The reports were inconsistent regarding the scope and the HTA assessment criteria. The link between HTA findings, HTA decision making, and health policies were not clear.

**Discussion:**

The inconsistencies between the reports and the methodological limitations demonstrate the need for national HTA guidelines. Improving the characteristics of the HTA might impact implementation. Among the key issues is transparency regarding priority setting, the HTA process, and decision making.

**Conclusion:**

Establishing and adopting national HTA guidelines at international standards is needed. Involving external scientific committees and health economists in the HTA processes might help ensure that the key principles of HTA are followed. The study findings might be helpful for countries that are developing their HTA systems.

## Background

The World Health Organization (WHO) estimates that between 20 percent and 40 percent of healthcare expenditures are wasted globally (1). Health technology assessment (HTA) is an essential component of developing evidence-based health policies, and it is gaining momentum in low- and middle-income countries (LMICs) to inform priority setting better and ensure that the limited resources are utilized optimally (2). HTA offers a systematic approach for decision makers to evaluate the clinical impacts and cost-effectiveness of health technologies and consider broader issues, such as health inequalities (3).

Budget constraints combined with a rise in demand increase the importance of resource allocation and evidence-based decision making in health care. Producing HTA reports and improving their implementations is one of the strategies included in the strategic plans published by the Turkiye Ministry of Health (MoH) for 2013–2017 and 2019–2023 to provide effective, efficient, and quality delivery of healthcare services (4).

The HTA agency of the Turkiye MoH was established in 2012, which aimed starting the transition from strategy to implementation (4). The agency was later named Research Development and Health Technology Assessment Department (HTAD) as part of the Health Services General Directorate under the MoH and is responsible for conducting evaluations of health technologies regarding effectiveness, efficacy, and efficiency. The agency’s HTA policy is defined as contributing to the financial sustainability of healthcare resources, encouraging the adoption of novel or neglected health technologies and reducing the use of clinically ineffective and financially unsustainable technologies. The agency works in collaboration with EUnetHTA and contributes to World Bank funded projects. There are two other national agencies that conduct HTAs in the country, the Agency for Pharmaceuticals and Medical Devices (TITCK) and the HTA Department within the Social Security Institution (SSI). TITCK acts a regulatory, supervisory, and advisory institution regarding pharmaceuticals, medical devices, traditional herbal and supportive products, advanced therapeutic medical products, and cosmetic products. It conducts pharmacoeconomic evaluations and studies to determine medicine prices. The HTA Department within SSI conducts economic analyses to inform reimbursement decisions of SSI which is the largest and sole public health insurance provider in the country. Turkiye’s national HTA agencies lack mandatory power, and it is not clear, how the agencies provide recommendations to the Social Security Institution (SSI) that makes the reimbursement decisions or policy makers. It is not clear, how these agencies based under different organizations interact with each other as there is no known formal mechanism for that. The country does not have publicly available, detailed national guidelines, but the MoH HTA Directive sets out the process of undertaking HTA, which applies to all the HTA activities undertaken by HTAD and TITCK (5).

The Smart Life and Health Products and Technologies Roadmap published by the Turkiye Ministry of Industry and Technology recommends establishing a politically independent national HTA institution between 2022 and 2025, which would provide objective scientific advice, similar to NICE in the UK and HAS in France (6).

The strategy document prepared by HTAD for 2019–2023 lists compliance with the key HTA principles developed by Drummond as a strategy for the institutionalization of HTA in the country (4;7). These principles described elements of good practice regarding HTA, consisting of fifteen items grouped by four aspects (i) structure of HTA; (ii) methods of HTA; (iii) processes for conduct of HTA; and (iv) use of HTAs in decision making. Given that 2022 marked the first decade of the country’s HTA journey and the establishment of a new independent interagency HTA body is underway, a review is timely (6).

The current study aimed to provide an overview of the country’s HTA progress since establishing the HTA agencies a decade ago, critically reviewing the MoH HTA Directive and the national HTA reports that are publicly accessible. This will contribute to improving the future national HTA activities and inform researchers and policy makers in Turkiye and other countries that are developing their HTA systems.

## Methods

The official web sites of the three national HTA agencies were searched in June 2022 and updated in November 2022. One reviewer completed the data extraction, and the second reviewer checked it for consistency, using a pre-piloted data extraction tool (Supplementary Material). The MoH HTA Directive was evaluated based on compliance with the key HTA principles developed by Drummond et al., whereas the HTA reports were assessed against the MoH Directive. The principles published by Drummond et al. were chosen as an assessment tool because the agency’s strategy document referred to them as guiding principles (5;7). The principles are non-prescriptive, flexible, and appropriate for HTA in different settings. The recommendations in the MoH Directive were grouped in line with the four key aspects in the Drummond’s principles; (i) structure; (ii) methods; (iii) processes for conduct; and (iv) use of HTAs in decision making. Two researchers independently conducted the assessment, and any discrepancies were dissolved through discussion.

## Results

**Figure 1.**
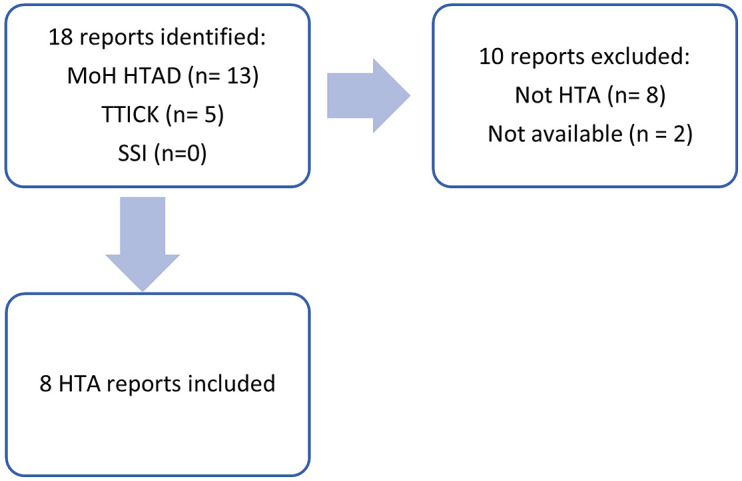
HTA reports selection process.

Figure 1 summarizes the report identification process. The web site search yielded eighteen reports titled as HTAs. Among these, five were published by the Turkish Medicines and Medical Devices Agency (TITCK), and these focused on market price changes of frequently used medications in the country and budget impact analysis with no evaluation of clinical effectiveness. Thus, they were not considered as HTAs in parallel with the definition that was agreed by international HTA agencies (3). There were thirteen reports published by HTAD: two international reports that the agency contributed, one clinical guideline wrongly titled HTA (8), and ten national HTA reports. Amongst these national HTA reports, eight were publicly available online, and the remaining two reports were not included because of not being publicly available. Through informal communication, it was understood that these reports were not published on the web site due to including commercially sensitive information submitted by companies. The study included all the available national HTA reports (9–16). As there no HTA report by SSI was identified, SSI was contacted, and they confirmed that no HTA report has been published to date.

### The MoH HTA Directive

The MoH HTA Directive was published in 2013 and updated in 2019, identifying the HTA steps and processes undertaken by the Turkiye MoH (5). In the first section, the Directive provides the terms and definitions, followed by a section which sets out the details of topic selection and the topic selection committee. The third section includes information on the HTA project process, including the conduct of the project and brief guidance on the literature search.

### The MoH HTA Directive against the Drummond’s principles

The overall compliance of the MoH Directive with the Drummond’s key principles was poor as only around one third of the principles were included (Table 1).

**Table 1.** Assessment of the HTA Directive and HTA reports against Drummond’s key principles of HTA (7)

Key principles of HTA	Directive included
*Structure of HTA*	
1. Was the goal and scope of the HTA explicit and relevant to its use?	Partly
2. Was the HTA an unbiased and transparent exercise?	No
3. Did the HTA report include all relevant technologies?	Partly
4. Was there a clear system for setting priorities for HTA?	Partly
*Methods of HTA*	
5. Did the HTA incorporate appropriate methods for assessing costs and benefits?	Partly
6. Did the HTA consider a wide range of evidence and outcomes?	Yes
7. Was a full societal perspective considered?	No
8. Was uncertainty surrounding estimates explicitly characterized?	No
9. Were issues of generalizability and transferability considered and addressed?	No
*Processes for conducting HTA*	
10. Were all key stakeholder groups actively engaged?	Yes
11. Have all available data been sought actively by those undertaking HTA?	Partly
12. Have the implementation of HTA findings been monitored?	Yes
*Use of HTA in decision making*	
13. Was the HTA published before the implementation of the technology?	No
14. Were the HTA findings communicated appropriately to different decision makers?	Partly
15. Was the link between HTA findings and decision-making processes transparent and clearly defined?	No
*Overall compliance with the principles*	33%

#### Structure of HTA

The Directive defines HTA based on the EUnetHTA core model as the systematic evaluation of health technologies in terms of their properties, safety, clinical effectiveness, efficiency, and economic, social, organizational, ethical, and legal aspects (17). The Directive states that the HTA reports can be published in the form of full HTA reports, rapid effectiveness studies, or adaptation studies. Full HTA reports should include all the EUnetHTA core model components, whereas rapid HTA reports are not defined. Adaptation studies refer to the translation and adaptation of HTA reports published by other HTA agencies. The Directive recommends the use of Population Intervention Comparator and Outcomes (PICO) approach to define the policy question as part of the HTA project process.

The Directive did not include explicit guidance on ensuring transparency and reducing bias in the HTA processes. There was no requirement to involve an external committee or any kind of peer-reviewing into the HTA process in the Directive.

It is essential to include all the relevant technologies in an HTA within the remits of an HTA agency, including standard care and, in some cases, no intervention as the comparator if there is no treatment available, and any exclusions should be justified. The Directive includes no statement on including all relevant technologies, leaving this at the discretion of the topic selection committee.

The Directive requires topic selection for HTA projects to be undertaken by a committee of stakeholders, including the MoH HTAD employees, academics, and civil society members. The committee decisions should be based on a set of criteria: (i) burden of disease, (ii) resource impact, (iii) public interest, and (iv) availability of qualified human resources to undertake the HTA on a specific topic. The committee decisions should be published on the agency web site along with the justifications for topic selections. Apart from the topics selected by the committee, the director of the agency can commission an HTA subject to the approval of the Health Services General Directorate, based on Article 7 of the Directive. It is not clear, however, whether the same criteria would apply when the topics are selected by the agency director and not by the committee.

#### Methods of HTA

The Directive provides very little guidance on methodological issues. It includes instructions for the database search. However, no specific method is named, and no guidance is provided on the evaluations. It does not include any guidance on dealing with uncertainty, generalizability, and transferability although it allows conducting adaptation studies based on HTA reports from other countries.

The primary healthcare provider and the funder of the healthcare services are two different bodies in the country that are the MoH and SSI, respectively. Hence, the perspectives of the two might differ, and it is crucial to define the perspective of the HTA explicitly. However, there is no mention of perspective in the Directive.

#### Processes for conduct of HTA

Engaging all key stakeholders is one of the key recommendations of the Directive, which lists the key stakeholders amongst the members of the topic selection committee. The Directive requires a “feedback period” of 15 days after the initial report is published, and based on feedback received during this period, the final report should be published after revisions. There is no statement on how information submitted by the stakeholders, including confidential data, should be handled.

The Directive requires the literature review to be conducted in a way that would enable identifying a wide range of evidence. It states that quality assessment should be undertaken based on the internationally accepted measures without naming any recommended tools.

#### Use of HTA in decision making

It is essential that HTAs are timely in terms of the opportunity to influence decision making, and HTA reports published after the implementation of a technology have limited potential to affect policy. The Directive does not include any recommendation in terms of the timing of HTA. To make sure the HTAs are up to date with the technological improvements and available data sets, the HTA agencies might review and update the published reports. The Directive requires the national HTA agency to review and update the reports when necessary. However, no timeline was stated, and it was not clear how “necessity” would be defined.

The findings of HTA reports are not always translated into implementation, and it is vital to understand the reasons behind that. This would enable agencies to improve the reports and promote greater implementation of the HTA evidence. The Directive states the implementation of HTA findings would be monitored, and challenges to implementation would be identified although the HTA reports are advisory only. However, no further details are provided either in the Directive or on the agency web site. Thus, the link between the HTA reports and decision making, and implementation is unclear.

### The HTA reports against the MoH Directive

The characteristics of the included HTA reports are presented in Table 2, and more details are provided in the Supplementary Material. The reports focused on various topics, including fetal anomalies, cancer, and obesity. The decision maker was not stated in any of the reports, and none was peer-reviewed. A summary of the assessment of the HTA reports against the HTA Directive is provided in Table 3. On average, the HTA reports showed a moderate level of compliance with the MoH Directive, ranging between 48 percent and 67 percent.

**Table 2.** Characteristics of the included HTA reports

Report no.	First author and year	Disease area	Intervention	Comparator	HTA core model domains	Economic evaluation conducted	Any guidelines followed	All stakeholders involved	Advisory committee/peer-review	Decision maker	Key policy advice
1	Tecirli* 2020(16)	Fetal chromosomal anomalies	Combined test (CT) only	CT + triple test, CT + quadruple test, CT + cfDNA	First six domains	Cost analysis	None	No	No/No	Not stated	Pathway change: Combined test should be prioritized, and the triple and quadruple tests should not be conducted for those who took the combined test.
2	Arslan 2019(15)	Sepsis	Molecular rapid tests + blood culture test	Blood culture test	First five domains	Cost-utility	None	No	No/No	Not stated	Molecular rapid tests for sepsis + blood culture test is more cost-effective than blood culture alone.
3	Mahagaonkar 2019(9)	Rheumatoid arthritis	bDMARDs	csDMARDs	All nine domains	Cost analysis	PRISMA	Yes	No/No	Not stated	Unclear
4	Kockaya 2018(10)	Cancer	Cytoreductive surgery + hyperthermic intraperitoneal chemotherapy	Cytoreductive surgery alone	First five domains	No	None	Yes	No/No	Not stated	Unclear
5	Ozturk 2018(12)	Male circumcision	Single-use devices that require minimal surgical implementation	Traditional surgery	All nine domains	Cost analysis	None	Yes	No/No	Not stated	Unclear
6	Gunal 2017(13)	Renal insufficiency	Peritoneal dialysis	Hemodialysis	All nine domains	Cost-utility	None	Yes	Yes/No	Not stated	Pathway change: Patients should start with peritoneal dialysis.
7	Sener* 2014(14)	Obesity	Surgery for obesity	No surgery	All nine domains	Cost analysis	None	No	No/No	Not stated	Unclear
8	Karadayı 2013(11)	Erectile dysfunction	Electroshock wave treatment (ESW)	Usual care (not defined)	First two domains	No	None	No	No/No	Not stated	Do not adopt: ESW should not be provided as part of the national healthcare system

* Project coordinator; authors were not stated.

**Table 3.** Assessment of the HTA reports against the HTA Directive

		HTA report number							
	HTA Directive statements	1*	2*	3	4*	5	6	7	8*
No.	*Structure of HTA*								
1	Topics selected by a committee of minimum seven members	?	?	?	?	?	?	?	?
2	Four considerations in topic selection by committee	?	?	?	?	?	?	?	?
3	Committee decisions are published on the web site with justifications	No	No	No	No	No	No	No	No
4	The goal of HTA and policy question is defined	Yes	Yes	Yes	Yes	Yes	Yes	Yes	Yes
	*Processes for conducting HTA*								
5	Two or more people are involved	Yes	Yes	Yes	Yes	Yes	Yes	Yes	No
6	One directorate member, universities, relevant public institutions, civil society members, and experts are involved as needed	No	Yes	Yes	Yes	Yes	Yes	Yes	No
7	Research questions and PICO are defined	Yes	Yes	Yes	Yes	Yes	Yes	No	No
8	Systematic literature review is undertaken	Yes	Yes	Yes	Yes	Yes	Yes	Yes	Yes
9	Quality assessment of the included studies is conducted, using international guidelines	No	Yes	Yes	No	No	No	No	No
10	EU and international terminology are utilized	Yes	Yes	Yes	Yes	Yes	Yes	Yes	Yes
11	Draft report is published on the web site for 15 days	?	?	?	?	?	?	?	?
12	Feedback from stakeholders is incorporated in 15 days	?	?	?	?	?	?	?	?
13	The final report is published on the web site	Yes	Yes	Yes	Yes	Yes	Yes	Yes	Yes
	*Methods of HTA*								
14	All HTA reports are published in Turkish	Yes	Yes	Yes	Yes	Yes	Yes	Yes	Yes
15	A lay version of all reports is published	Yes	Yes	Yes	Yes	Yes	Yes	Yes	Yes
16	International databases are searched, such as EMBASE	Yes	Yes	Yes	Yes	Yes	Yes	Yes	Yes
17	Keywords are defined to reach a wide evidence base	Yes	Yes	Yes	Yes	Yes	Yes	Yes	Yes
18	Clinical effectiveness literature review and the report are written by experts in the field	Yes	Yes	Yes	Yes	Yes	Yes	Yes	Yes
19	Economic, social, ethical, and legal evaluations are conducted by the project members who work in the relevant fields	No	No	Yes	No	Yes	Yes	Yes	Yes
	*Use of HTA in decision making*								
20	Implementation of the HTA reports is monitored	?	?	?	?	?	?	?	?
21	The HTA reports are updated if deemed necessary	?	?	?	?	?	?	?	?
	*Compliance*	52%	62%	67%	57%	62%	62%	57%	48%

* Defined as a short HTA report since the report did not include all nine domains of HTA core model.

? Information could not been obtained from the reports.

#### Structure of HTA

Although the Directive required committee decisions on HTA topic selections to be published on the web site, this was not available for any of the reports. Some reports mentioned conducting meetings with stakeholders to define the scope of the HTA (10;13); however, none provided a scoping document or a protocol, and these were not available in the reports or on the agency’s web site.

The goal of HTA was expressed in all but one report (11), using the PICO approach as recommended by the Directive. Only four reports included all nine domains of the Core HTA model (17), and the others omitted three or more domains with no justification (9;12–14).

Almost all the reports provided the blank conflict of interest form as appendix along with a statement confirming that all the contributors signed the document and declared no conflict of interest except three (11, 13, 15). Although its role was not defined, one of the reports included an external advisory panel (13), and two reports mentioned receiving feedback with no documentation on how the feedback was sought and changes to the report after receiving feedback (9 and 15). None of the remaining reports stated whether there were any efforts to increase transparency and reduce the risk of bias.

Although the Directive requires the agency to publish all topic selection decisions on the web site, none was published. The published HTA reports did not provide information on the topic selection process, except one which stated that the report did not go through the topic selection process described in the Directive (11). Thus, despite having a somewhat clear system for priority setting, the implementation of that system was not entirely transparent.

#### Methods of HTA

In the HTA reports, the costs and health benefits mainly were assessed based on systematic reviews. However, since the reviews did not follow any guidelines, there were significant limitations. For instance, some systematic reviews had time restrictions with no justifications (10;12), and hence, it is likely that some studies were missed. Most reports either did not include an economic evaluation or only included a cost analysis. Only two reports included a cost-utility analysis, but the sources of quality-adjusted-life-year (QALY) values were not reported in one (13), and the second included significant assumptions (15).

All the reports included observational studies as well as randomized controlled trials. Unexpectedly in one report, the methodology section stated that non-randomized studies were excluded although observational studies were included (9). None of the reports included qualitative studies. Some reports (*n* = 4) only focused on clinical outcomes, excluding other outcomes such as quality of life (11; 12; 15; 16).

Perspective was described in three HTA reports only (22). The economic evaluation conducted as part of one report clearly stated its perspective as the societal perspective and considered the productivity costs, social benefits provided to the patients who needed dialysis, as well as transportation costs (13). There was no discussion on the costs and outcomes that fall outside the healthcare system in the other reports. For example, the health and cost impacts on carers were not considered. Additionally, five reports discussed the implications around wider issues as part of the Core HTA model (9; 11–14). These included social and legal aspects, which were limited to patient and expert perspectives, patients’ rights and patent regulations. Health inequalities were only considered in terms of access (i.e., national health insurance coverage), and the relationship between inequities in society and the health conditions was not evaluated. Similarly, no evaluation was undertaken to understand whether the new technologies in the report contributed to reducing health inequalities.

Uncertainty was one of the most overlooked principles of HTA, being not mentioned in the Directive. Two reports addressed uncertainty explicitly, conducting a probabilistic sensitivity analysis (22). However, the potential impact of uncertainties on findings was not discussed, and the confidence intervals of the QALY and cost estimates were not provided.

There was limited and inconsistent consideration of the applicability of the evidence produced in other countries to Turkiye despite relying on international data for most analyses. It was also not clear which countries were considered more comparable to Turkiye. For instance, the report on rheumatoid arthritis limited the systematic review to Russia, Germany, France, the UK, USA, and Turkiye with no justification, and the same report mentioned the cost-effectiveness of antitumor necrosis factors in Japan, South Korea, and Taiwan, although the literature search did not include these countries (9).

#### Processes for conduct of HTA

Although some reports mentioned including all stakeholders (9;10;12), such as patients’ representatives and healthcare professionals, the methodological details were not provided. Only one HTA report mentioned using the Delphi method to gather views of ten experts and focus group meetings with two patients’ representatives (9). However, it was not clear how they were selected and whether they could be considered a representative group. The number of healthcare professionals and patients consulted was not provided in the remaining reports. It was not clear whether the Directive’s recommendation on seeking feedback from all stakeholders was implemented since there was no mention of such amendments in the published reports. The Directive requires identifying a wide range of relevant evidence. However, only one report included a gray literature search (12), and the systematic reviews had some restrictions regarding the type of the studies (9), timelines (10,12);, and settings (9) with no justification. The quality of the included studies was assessed only in two reports (9;15), although quality assessment is listed as part of the literature review process in the Directive.

Since the reports did not include much information on the overall HTA process, it was unclear whether companies submitted any data and how these were assessed. Some reports stated that companies contributed to the HTA projects, but it was not explained how they influenced the processes (17).

#### Use of HTA in decision making

Amongst the published HTA reports, none was reviewed and updated. Additionally, none of the reports discussed when or under what circumstances they should be updated.

Two HTA reports were not available online, although it is a requirement based on the Directive. Most reports provided a patients’ and carers’ summary in addition to the executive summary as recommended by the Directive except three.

The HTA reports published by the Turkiye MoH HTA agency were not tied to a particular decision, and hence, most reports did not include a clear policy recommendation. Since Turkiye currently does not have a national cost-effectiveness threshold, some reports compared the cost-effectiveness of the technologies under evaluation in different countries but refrained from discussing the policy implications specific to Turkiye. None of the reports provided any information about how the implementation would be monitored.

## Discussion

The study aimed to evaluate the first decade of HTA activities in the country based on the key principles outlined by Drummond et al. (7), reviewing the HTA Directive and the HTA reports published by the MoH HTA agency which is based under MoH. It was found that the HTA Directive did not cover many essential principles, such as perspective and dealing with uncertainty, and compliance with the key HTA principles was low to moderate in the HTAs conducted by Turkiye’s national HTA agency. There were inconsistencies in the reports regarding the scope and the assessment criteria. A key issue was transparency regarding priority setting, the HTA process and decision making. Most HTA reports were published online and made publicly available; however, those reports included little or no information on the broader process of HTA, such as the committee meetings and stakeholder involvement. It was also unclear how companies influenced the HTA process, although they were named as contributors in some reports. There were also significant methodological limitations in the published HTA reports.

### Strength and limitations

This is the first critical review of the national HTA reports published in Turkiye during the first decade of HTA. The findings of this study are limited to the information obtained from published national HTA reports, the National HTA Directive and the HTAD web site. There might be some other national HTA reports produced but not publicly available. Hospital-based HTA reports were not included in this study because the study focused on the HTAs conducted from a healthcare system perspective. Additionally, although the assessment of the HTA Directive and the published HTA reports is a good indicator of the HTA in the country, there might be some other aspects that are not considered in this study.

### Findings in the context of literature

The low levels of compliance with the key principles of HTA identified in this study contradicts the finding of a stakeholder survey that reported a good level of compliance in the country’s HTA processes (18). For example, the participants mostly thought that uncertainty was explicitly characterized, and a full societal perspective was considered. However, the Directive does not include any recommendations on these issues and only one of the HTA reports addressed uncertainty partly while no recommendation on perspective was provided in the Directive and the societal perspective was considered only in one report. This can be partly explained by the fact that the survey participants did not have access to the paper which explained the key principles in detail. Additionally, the participants had to rely on their subjective knowledge of the HTA processes at the time of the survey. A review of the HTA Directive and the HTA reports by two researchers independently enabled this study to provide a more systematic assessment of the HTA processes. A similar method was followed in a previous evaluation of international HTA practices (19).

The challenges of benchmarking HTA agencies have been discussed in the literature (20). It is important to acknowledge that following all the key principles of HTA developed by Drummond might not be possible for all HTA agencies due to, for example, limited resources available. Some principles might be more important than others for a given setting or project. Nevertheless, having a set of guidelines is key to ensuring good practice, and the limitations of the HTAs must be discussed explicitly in the reports. That would not undervalue the work conducted by the HTA agencies; on the contrary, would improve their credibility. The national HTA agency welcomes this approach by setting an aim to follow the key principles in the strategy document, and this is promising for the future of HTA in the country.

### Implications

Lacking mandatory power, it is challenging for HTA to influence decision making in Turkiye. Improving the characteristics of the HTA might impact implementation. This study identified some issues to be addressed to improve the HTA processes and the HTA reports and guide the future HTA activities in Turkiye and other countries that are developing their HTA structures.

The inconsistencies between the reports and the methodological limitations clearly demonstrate the need for national HTA guidelines. Until the national guidelines are established, the researchers can use guidelines of established HTA agencies, such as the NICE technology appraisal guidelines from the UK (21). Additionally, following the internationally recognized guidelines for the different sections of the HTA reports should be considered; including systematic review and economic evaluation guidelines.

The study raises some concerns over the consideration of transparency and bias in the HTA process. The decisions on topic selection have not been published despite the requirement in the National HTA Directive. It is important to follow clear priority setting criteria and make this publicly available. Additionally, to ensure that HTA is an unbiased activity, involving an external advisory panel for each HTA might be helpful. It is also important to be explicit about the role of the panel. Additionally, HTA reports might be subjected to peer-review by experts in the relevant fields, which would improve the methodological quality as well as reduce the impact of potential bias.

It has been suggested that the HTA organizations should be independent of the institutions that are responsible for adopting, funding, and implementing the HTA decisions. Being part of the MoH might be reducing the credibility of the agency and limiting the application of the HTA reports. Thus, it might be helpful to establish an independent body or introduce mechanisms to distance the HTA agency from the main healthcare provider (MoH) and payer (SSI) in the country. Thus, the recommendation by the Turkiye Ministry of Industry and Technology in the Smart Life and Health Products and Technologies Roadmap and the accompanying regulations are significant steps in the right direction.

The use of health economics methods in the HTAs has been limited. Recently, a significant number of health economics studies have been published by researchers in Turkiye, indicating an emerging health economics capacity. Establishing formal links and working groups in collaboration with the universities, especially those that have postgraduate degree programs in health economics, would be helpful to utilize the country’s existing human resources.

HTA reports should be able to go beyond just merely acknowledging the use of data from other countries as a limitation. The transferability and applicability of international evidence to the Turkiye setting should be assessed for the technologies that are being evaluated, using specific guidelines or qualitative studies (22). Regardless of the availability of national data, probabilistic sensitivity analysis in HTA is a fundamental part of HTA. However, only one of the included HTA reports included a probabilistic sensitivity analysis, and the implications regarding the policy advice were not discussed.

Although the National HTA Directive states that the agency would undertake adaptation of the HTA reports produced by other countries in addition to conducting national HTAs, no adaptation report has been published. Given that the published reports identified no or a limited number of studies from Turkiye, producing adaptation reports might be a feasible and more cost-effective option for the country. Agencies in many developed countries spend many resources on defining and assessing the available evidence. For example, the HTA reports published by NICE might be appropriate for these adaptation reports, considering the similarities between the healthcare systems. However, the standard guidelines for adaptation reports must be followed to ensure objectivity and consistency (23).

Another related consideration is the lack of a national willingness-to-pay threshold. This might be one reason why the HTA reports refrained from assessing or discussing cost-effectiveness. However, the GDP-based threshold previously recommended by WHO for LMICs was used in two reports included in this study (17) although WHO recently changed its stance on the use of this threshold as a decision rule (24). Thus, the HTA agency should clarify their recommendation on this issue.

Finally, it is important to identify impact objectives for HTAs in addition to the scientific goals, such as affecting reimbursement decisions or clinical practice. Gerhardus et al. (25) suggest a six-step approach to define and evaluate the impact of HTA activities regarding awareness of HTA reports, attitudes toward HTA reports, and impacts on health policy process, health policy decision, clinical practice, and health and economic parameters. For example, one study from Canada showed that following the HTA recommendations would save around 3 million Canadian dollars annually (26). Considering the impact that HTA is aiming to achieve might help formalize the role of HTA in healthcare decision making as well as guide future research.

### Conclusion

Turkiye’s progress in HTA has been slow, and a decade after establishing the first HTA agency, the role of HTA in healthcare decision making remains unclear. The study findings provide important learnings for the HTA agencies, researchers, and policy makers in countries where HTA is developing. The link between HTA findings and decision-making processes needs to be explicitly defined and formalized. There is an urgent need to establish and adopt national HTA guidelines that meet the international standards. Involving external scientific committees and health economists in the HTA processes might help ensure that the key principles of HTA are followed.

## Supporting information

Avşar and Yıldırım supplementary material Avşar and Yıldırım supplementary material

## Data Availability

All the reports in this study are available online.

## Supplementary material

The supplementary material for this article can be found at https://doi.org/10.1017/S0266462323000466.
