# Supplementary material for: National health technology assessment in Turkiye after a decade: are key principles followed?
Source: Int J Technol Assess Health Care. 2023 Jul 24;39(1):e52. doi: 10.1017/S0266462323000466 (PMC11570135; doi:10.1017/S0266462323000466)
Supplement: Avşar and Yıldırım supplementary material [file S0266462323000466sup001.docx]

Electronic Supplementary Material

## National Health Technology Assessment in Turkiye after a decade: Are key principles followed?

Tuba Saygın Avşar^1^, Hasan Hüseyin Yıldırım^2^

^1^ Department of Applied Health Research, University College London, London, UK, ORCID ID: 0000-0002-4143-3852

^2^ Department of Health Management, University of Health Sciences, Ankara, Turkey, ORCID ID: 0000-0002-3598-7031

Corresponding author: [t.avsar@ucl.ac.uk](mailto:t.avsar@ucl.ac.uk)

Table 1. Additional information on the HTA reports

| **First author & year** | **Disease**  **area** | **Topic selection** | **Number of people with the condition** | **Executive & lay summary provided** | **Contributions statement** | **Conflict of interest** | **Comparator and intervention defined clearly** | **Systematic review conducted** | **Details of SR provided (e.g. keywords, list of studies)** | **Quality assessment** | **Meta-analysis conducted** | **How is cost-effectiveness defined? (e.g. threshold)** | **Perspective and price year defined** | **Uncertainty addressed** | **Applicability of evidence from other settings discussed** | **Details of stakeholder involvement provided** | **Health inequalities considered** | **Affordability considered** |
| --- | --- | --- | --- | --- | --- | --- | --- | --- | --- | --- | --- | --- | --- | --- | --- | --- | --- | --- |
| Tecirli  2020 | Fetal chromosomal anomalies | No details provided | 1.3m. deliveries annually | Yes/Yes | No | None declared | Partly | Yes | Partly | No | No | Not defined | No/No | No | No | Not applicable | No | No |
| Arslan  2019 | Sepsis | No details provided | 17% of all patients in intensive care | Yes/Yes | Yes | None declared | Yes | Yes | Yes | QUADAS-2 | Yes | 3 times GDP | Yes/Yes | Yes | Partly | Not applicable | No | No |
| Mahagaonkar  2019 | Rheumatoid arthritis | No details provided | 351,789 | Yes/Yes | No | None declared | Yes | Yes | Partly | AMSTAR | No | Not defined | No/No | No | Partly | Partly | No | No |
| Kockaya  2018 | Cancer | No details provided | Not reported | Yes/Yes | No | None declared | Yes | Yes | Partly | No | No | Not defined | Not applicable | No | No | No | No | No |
| Ozturk  2018 | Male circumcision | No details provided | 418,283 males in 2017 | Yes/Yes | Yes | None declared | Yes | Yes | Partly | No | No | Not defined | No/No | No | Partly | No | No | Partly |
| **Gunal**  2017 | Renal insufficiency | No details provided | 73,660 patients needed dialysis in 2015 | No/No | Yes | None declared | Yes | Yes | Yes | No | No | 3 times GDP | Yes/No | Partly | Partly | No | No | Yes |
| **Sener***  2014 | Obesity | No details provided | 17% of the population was obese in 2012 | No/No | Yes | Not included | No | Yes | Partly | No | No | Not defined | Yes/No | No | Partly | Not applicable | No | Yes |
| **Karadayi 2013** | Erectile disfuntion | No details provided | Not reported | No/No | No | No statement | No | Yes | No | No | No | No | Not defined | Not applicable | No | No | Not applicable | No |
| *Project coordinator; authors were not stated. | | | | | | | | | | | | | | | | | | |
